# Successful hot balloon ablation for focal atrial tachycardia derived from the left superior pulmonary vein antrum

**DOI:** 10.1002/joa3.12313

**Published:** 2020-02-14

**Authors:** Hiroaki Tabata, Ayako Okada, Hideki Kobayashi, Wataru Shoin, Takahiro Okano, Koji Yoshie, Hirohiko Motoki, Morio Shoda, Koichiro Kuwahara

**Affiliations:** ^1^ Department of Cardiovascular Medicine Shinshu University School of Medicine Matsumoto Japan

**Keywords:** atrial fibrillation, atrial tachycardia, catheter ablation, hot balloon, supraventricular tachycardia

## Abstract

A 45‐year‐old man with paroxysmal atrial fibrillation (PAF) was admitted to our hospital for hot balloon ablation. At admission, atrial tachycardia (AT) was observed. Activation map showed focal atrial tachycardia originating from the posterior wall of the left superior pulmonary vein (LSPV) antrum. We performed hot balloon ablation at the LSPV antrum, terminated AT, and performed pulmonary vein isolation with a hot balloon. The hot balloon was successfully applied for the ablation of the focal atrial tachycardia from the pulmonary vein.

## Graphical abstract

Hot balloon was successfully applied for the ablation of the focal atrial tachycardia from the left superior pulmonary vein.

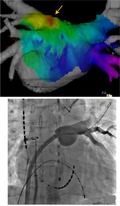

## 1 INTRODUCTION

The radiofrequency hot balloon catheter was developed for the ablation of paroxysmal atrial fibrillation (PAF). Although its efficacy in pulmonary vein isolation (PVI) has been established, its utilization for the ablation of other types of supraventricular tachycardia has not been reported. We describe a case of successful hot balloon ablation for focal atrial tachycardia derived from the left atrium.

## 2 CASE REPORT

**Figure 1:**
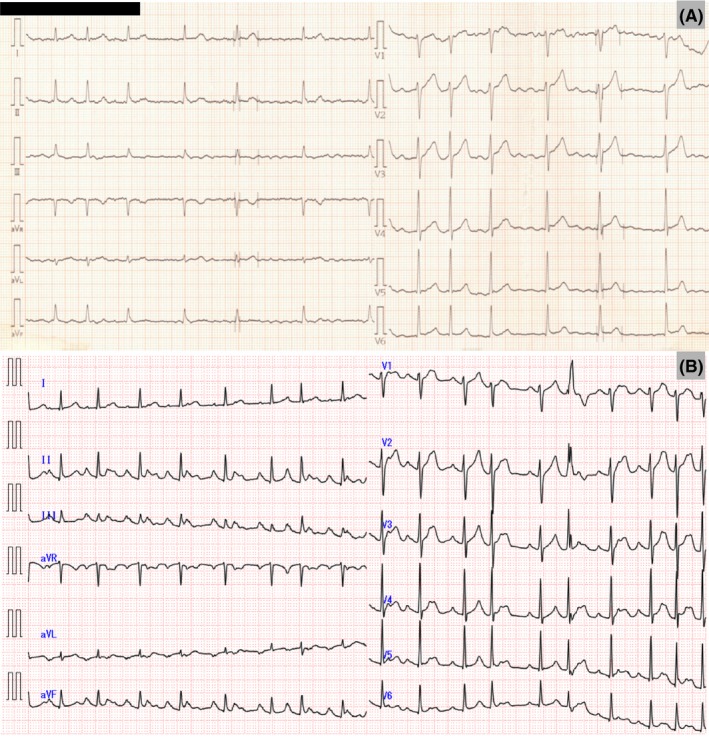
A, Electrocardiogram showing paroxysmal atrial fibrillation. B, Electrocardiogram at admission showing atrial tachycardia. P waves were positive in leads V1, Ⅱ, Ⅲ, and aVF, and negative in lead aVL

A 45‐year‐old man presented with paroxysmal palpitation to the Shinshu University Hospital. Electrocardiogram revealed PAF (Figure 1A), and the patient chose hot balloon ablation because of drug‐resistant palpitations. Echocardiography showed no valvular heart disease, a left atrial volume index (LAVI) of 26.9 mL/m^2^, and a 65% left ventricular ejection fraction based on the modified Simpson method.

At admission, persistent atrial tachycardia (AT) was observed (Figure 1B). During AT, the P waves were positive in lead V1, negative in lead aVL, and positive in leads II, III, and aVF with high amplitude, indicating that the AT originated from the left superior pulmonary vein (LSPV) or the left atrial appendage.

**Figure 2:**
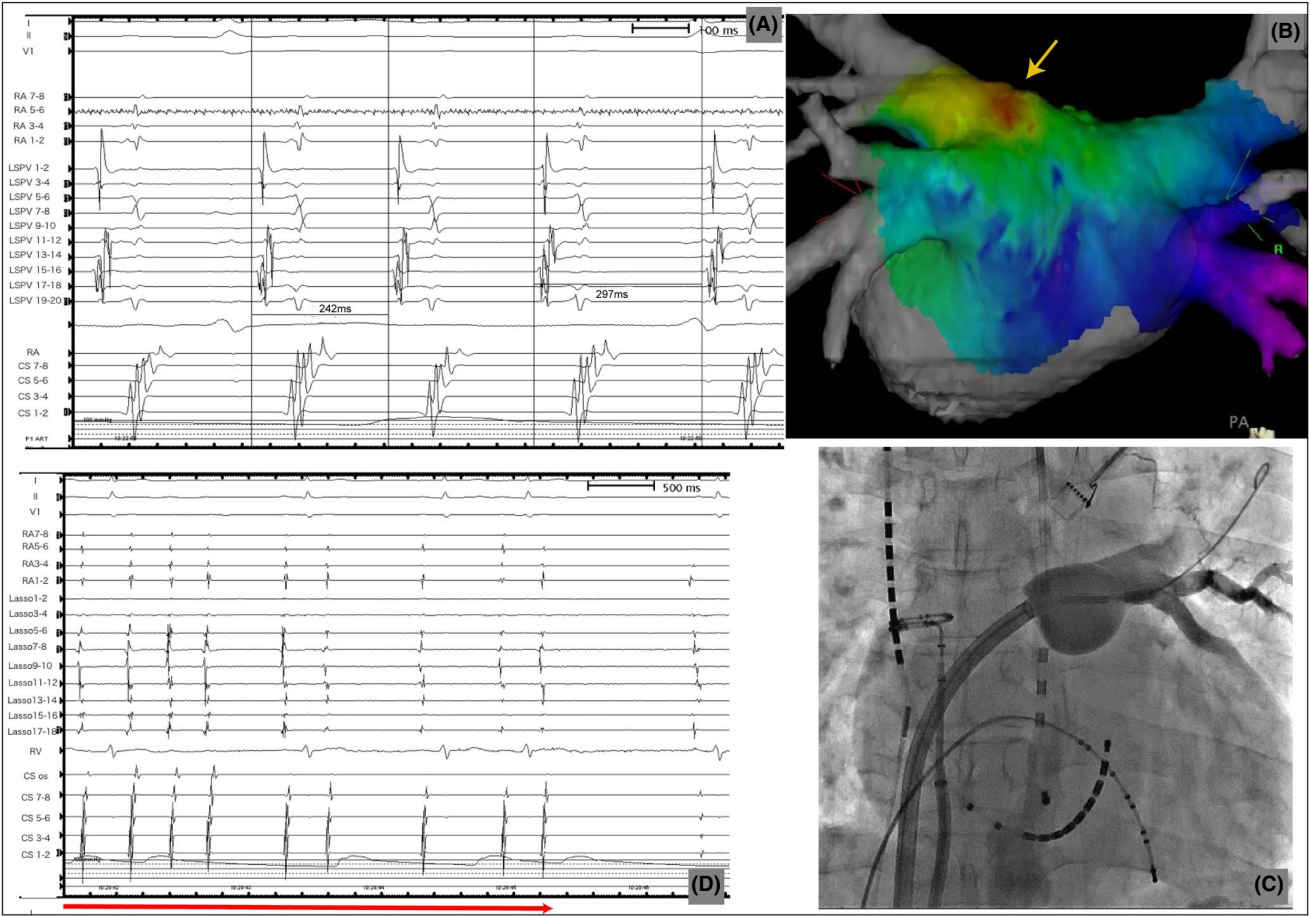
A, The intracardiac electrocardiogram during atrial tachycardia (AT). A‐A intervals varied beat by beat. B, CARTO activation map of AT. The source of focal AT was the posterior wall of the left superior pulmonary vein (LSPV) antrum (arrow). C, Venogram showed good occlusion of the LSPV by the balloon. D, AT was terminated 5 s from the start of ablation (arrow). Lasso catheter was placed at the posterior wall of the left atrium

The procedure was performed during AT. Although the A‐A intervals varied beat by beat from 242 to 297 ms, the atrial activation sequence was constant (Figure 2A). By the transseptal approach, an activation map of the left atrium was obtained using CARTO (Biosense Webster, Inc) and PentaRay catheter (Biosense Webster). The activation map showed that the focus of the early activation of AT was on the posterior wall of the antrum of the LSPV (Figure 2B). It seemed that the focus of the AT was exactly on the ablation site of the hot balloon at the LSPV antrum. We inflated the hot balloon with 10 mL of the contrast media that was diluted 1:2 by saline at the LSPV antrum. Venogram showed good occlusion of the LSPV by the balloon, and we started ablation. AT was terminated 5 seconds from the start of the ablation, and sinus rhythm was restored. We continued the ablation for 180 seconds at 70°C. During ablation, cold saline was injected into the esophagus, such that the temperature would not exceed 39°C. Then, we proceeded with the PVI by ablating other pulmonary veins with the hot balloon in an ordinal manner. We confirmed noninducibility of any AT or AF by burst atrial stimulation and finished the procedure. After discharge, there has been no recurrence of AT or AF.

## 3 DISCUSSION

This is the first report of a successful hot balloon ablation for focal atrial tachycardia derived from the left atrium. Cryoballoon ablation for focal atrial tachycardia from the left atrial appendage has been reported before,^1^ but hot balloon ablation for focal left atrial tachycardia has not been attempted until now.

The Toray‐Satake HotBalloon (TORAY Industries, Inc) was developed for catheter‐based ablation of paroxysmal AF. The AF‐free rate of hot balloon ablation has been reported to be 59% after 9 months of follow‐up,^2^ comparable to that of the traditional radiofrequency or cryoballoon ablation.

One unique feature of the HotBalloon is its high compliance that makes it possible to fit to the ostium of any size or any form of the pulmonary vein. Even patients with large left common pulmonary veins can undergo PVI with hot balloon ablation.^3^ In the present case, the patient developed not only AF but also AT, and hot balloon ablation was effective for both arrhythmias because the AT originated from the LSPV antrum that could completely fit the HotBalloon. The variation in A‐A intervals and centrifugal pattern of the activation map indicated that this AT was the focal AT. Although the moment of AF induction from this AT has not been recorded, focal AT originating from the PV could have contributed to the induction and maintenance of AF in this middle‐aged patient with a normal heart.

Pulmonary veins are known to be the most frequent origins of the focal left AT. Hachiya described that 46% (24/52 cases) of focal left ATs originate from the ostium of a pulmonary vein and that the superior PV is a more frequent origin than the lower PV (20 cases vs 4 cases).^4^ For the treatment of focal AT with a PV origin, single PVI has been reported to be superior to focal ablation of the source of AT, in terms of recurrence.^5^ Thus, as in this case, high‐compliant hot balloon ablation could be a promising treatment option for focal AT from any type of PV.

## CONFLICT OF INTERESTS

The authors declare no conflict of interest for this article.
